# Implementation of HybridArc treatment technique in preoperative radiotherapy of rectal cancer: dose patterns in target lesions and organs at risk as compared to helical Tomotherapy and RapidArc

**DOI:** 10.1186/1748-717X-7-120

**Published:** 2012-07-31

**Authors:** Thierry Gevaert, Benedikt Engels, Cristina Garibaldi, Dirk Verellen, Peter Deconinck, Michael Duchateau, Truus Reynders, Koen Tournel, Mark De Ridder

**Affiliations:** 1 Department of Radiotherapy, Universitair Ziekenhuis Brussel, Vrije Universiteit Brussel, Brussels, Belgium; 2 Department of Medical Physics, European Institute of Oncology (EIO), Milan, Italy

**Keywords:** HybridArc, Helical Tomotherapy, RapidArc, Simultaneous integrated boost, Preoperative radiotherapy, IMRT rectal cancer

## Abstract

**Purpose:**

HybridArc is a novel treatment technique blending aperture-enhanced optimized arcs with discrete IMRT-elements, allowing selection of arcs with a set of static IMRT-beams. This study compared this new technique to helical Tomotherapy, and RapidArc, in preoperative radiotherapy of rectal cancer.

**Material and methods:**

Twelve rectal cancer patients treated consecutively with Tomotherapy Hi-Art II system were simulated with HybridArc and RapidArc. Treatment plans were designed to deliver homogeneous dose of 46.0Gy to mesorectum and draining lymph nodes, with a simultaneous-integrated-boost to the primary tumor up to a total dose of 55.2Gy. Planning objectives were 95% of prescribed dose to 95% of PTVs, while minimizing the volume of small bowel receiving more than 15Gy (V15) and the mean bladder dose. Dose gradient towards simultaneous-integrated-boost (GI), calculated by dividing the volume receiving more then 52.4Gy (95% of PTV55.2Gy)to the volume of PTV55.2Gy, was kept below 1.5. Mean beam-on time and amount of MUs were also analyzed.

**Results:**

PTV swere adequately covered by all plans. Significant advantage was found for Tomotherapy in sparing small bowel (V15 = 112.7cm^3^SD73.4cm^3^) compared to RapidArc (133.4cm^3^SD75.3cm^3^) and HybridArc (143.7cm^3^SD74.4cm^3^) (p < 0.01). The mean bladder dose was better with RapidArc (20.6GySD2.2Gy) compared to HybridArc (24.2Gy SD4.3Gy) and Tomotherapy (23.0GySD4.7Gy) (p < 0.01). The mean beam-on time was significantly lower (p < 0.01) for HybridArc (2.7min SD0.8) and RapidArc (2.5min SD0.5) compared to Tomotherapy (11.0min SD0.7). The total amount of MUs was significantly (p < 0.01) lower for RapidArc (547SD44)compared to HybridArc (949 SD153).

**Conclusions:**

HybridArc is a feasible solution for preoperative RT with a simultaneous-integrated-boost in rectal cancer patients. It achieved similar PTV coverage with significant lower beam-on time, but less efficient in sparing small bowel and bladder compared to Tomotherapy and RapidArc. The added value of HybridArc is that the treatment modality can be implemented on every LINAC equipped with Dynamic-Conform-Arc and IMRT treatment techniques, while maintaining the same QA-schemes.

## Introduction

In patients with locally advanced rectal cancer, preoperative (chemo) radiotherapy (RT) is considered standard of care by improving local tumor control and overall survival [1-4]. The German Rectal Cancer Study Group reported any grade 3 acute and late toxicity in 27% and 14% of the patients undergoing conventional RT, respectively [5]. The relative large horseshoe-shaped planning target volume (PTV), with the bladder and small bowel lying in the middle, justifies the use of intensity-modulated RT (IMRT). Previous studies in our department demonstrated the advantages of IMRT and image-guided RT (IGRT) by helical Tomotherapy, regarding accurate dose delivery, minimization of the setup margin, normal tissue sparing and toxicity [6-8].

Intensity-modulated arc therapy (IMAT), as described by Yu [9], uses multiple treatment arcs with continuous modulation of the shape of the treatment fields by a multileaf collimator (MLC). RapidArc is a Volumetric modulated arc therapy (VMAT) delivery technique and hence an evolution of the IMAT concept. The treatment is delivered in arc rotation during which the MLCs are moving dynamically while the dose rate and gantry speed are also varying [10,11]. HybridArc is novel treatment technique and a different interpretation of the IMAT concept, blending aperture-enhanced optimized arcs with discrete IMRT-elements. This method allows selection of Aperture-optimized-Arcs, delivered with constant gantry speed and dose rate, with a set of static IMRT-elements at specified intervals along each arc. By weighting the contribution of arcs versus IMRT-elements, HybridArc aims achieving an optimal dose distribution, while limiting the use of IMRT.

Since HybridArc should provide a new approach of IMRT and IMAT, it is critical to assess its performance on a theoretical basis before clinical implementation. Because of the clinical meaningfulness of using IMRT in preoperative RT of rectal cancer, this study was designed to evaluate this new treatment technique in the neo-adjuvant setting of rectal cancer patients and to compare HybridArc to RapidArc and helical Tomotherapy.

## Material and methods

### Patient and treatment characteristics

For this study, we selected 12 consecutive patients with stage II/III rectal cancer treated preoperatively in our department in a phase II study with the Tomotherapy Hi-Art II system (Tomotherapy Inc., Madison, WI). According the protocol, all patients received a dose of 46.0Gy, in daily fractions of 2.0Gy, to the mesorectum and draining lymph nodes. Patients with a circumferential resection margin (CRM) of less than 2 mm on magnetic resonance imaging (MRI) received a simultaneous-integrated-boost to the primary tumor of 0.4Gy/day. Delineation of the CTV and OARs, CTV-PTV margins and values of planning parameters for helical Tomotherapy were previously extensively described [6]. Briefly, the planning goals were delivering at least 95% of the prescription dose to 95% of the PTV46.0Gy and PTV55.2Gy, while keeping the normalization to the mean dose of both PTVs and the irradiated volume of small bowel and mean bladder dose as low as possible. As the volume of small bowel receiving more than 15Gy (V15) is a known significant volumetric factor for developing grade 3+ acute small bowel toxicity [12], this parameter was used for inverse planning [13]. The 12 patients were simulated on the RapidArc treatment planning system Eclipse v8.6 (Varian, Palo Alto, CA) and on the iPlan v4.5 (Brainlab AG, Feldkirchen, Germany) treatment planning system, where a preclinical version of a new treatment modality HybridArc was installed.

### HybridArc treatment modality

HybridArc is a technique, which automatically blends the Aperture-optimized-Arcs with discrete IMRT technology. The arcs are inversely optimized Dynamic-Conformal-Arcs (DCAs), where the MLC shapes can be automatically modified to respect predefined objectives for the PTV and OARs. The automatic aperture optimization replaces elaborate and time-consuming manual field shaping and generates consistent and user-independent results. A classic DCA treatment employs leaf adaptations during the arc movement, while keeping the gantry speed and dose rate constant. The software typically uses control points with a 10° gantry spread step size, to determine a MLC planning shape specific to the projection of the PTV in this beam direction. The MLC leaves adapt to the different planning shapes considering the machine specific constraints of the MLC leaf movements. The HybridArc aperture optimization technique modifies classic DCA planning shapes to fulfill predefined PTV and OAR objectives. The aperture optimization is similar to optimizing several co-planar IMRT-elements: first beam fluences are optimized, and then these fluences are transformed to leaf patterns. During an aperture optimization, the classic dynamic conformal beam shape of each control point is subdivided into arc specific fluence beamlets. The optimization algorithm determines, in considering all other control points, the optimal fluence pattern for each single control point to fulfill the desired constraints. Unlike IMRT fluence patterns, the aperture optimization fluence uses only two fluence beamlet levels: fully irradiated, and not irradiated at all. The resulting control points fluence pattern is used to generate its specific planning shape.

The inverse planning engine for the aperture optimization process uses a combination of a Maximum-Likelihood-Estimator with dynamically changing penalization, resulting in the Dynamically-Penalized-Likelihood algorithm [14]. This algorithm has been introduced successfully in iPlan’s static IMRT-beam optimization and has been adapted for the aperture optimization process.

In other words, the method can be considered as a combination of forward planning (the number and position of the arcs as well as the start and stop angles, and the number of IMRT-elements are user defined) and inverse planning to optimize the aperture of the MLC from an anatomical shape of the target with or without excluding the critical structures. This Aperture-optimized-Arcs step is fully automated and can be guided by various penalization parameters to achieve appropriate OAR protection (dose optimization for OAR sparing at the cost of PTV coverage) including MU efficiency in the optimization process. After completion of the Aperture-optimized-Arcs, the inverse planning concentrates on the intensities in the IMRT-elements by moving the leaves continuously during each beam, to boost up the PTV coverage and to achieve a volumetric dose painting of the PTV within the provided dose constraints for the OAR. The user can adapt the weighting between Aperture-optimized-Arcs and discrete IMRT-elements prior to the optimization.

### HybridArc planning

**Figure 1:**
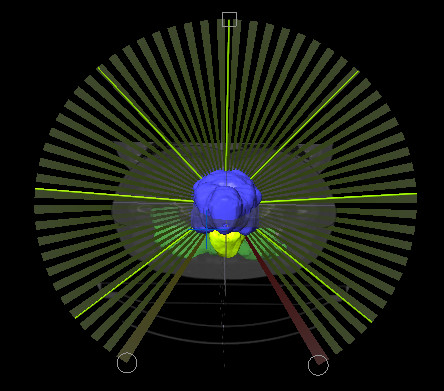
One Aperture optimized Arc with a gantry start/stop of 200°-160° with 7 discrete IMRT elements equally distributed over the arc.

HybridArc plans were generated for a Trilogy (Varian, Palo Alto, CA) linear accelerator (Linac), which is equipped with a dynamic high-resolution-MLC (Millennium 120 leaves MLC) characterized by a spatial resolution of 5 mm at isocenter for the central 20 cm and of 10 mm in the outer 20 cm). In this study, we used one Aperture-optimized-Arcs with a gantry start/stop angle of 200°-160° and 6-7 discrete IMRT-elements equally distributed over the arc (Figure 1), with a weight of 60% and 40% for Aperture-optimized-Arcs and IMRT, respectively. The planning constraints for the Aperture-optimized-Arcs were medium OAR sparing, which will exclude half of the overlap volume of the OAR within the PTV. Once the arc optimized, the IMRT-elements will accomplish the PTV coverage and the horseshoe shape of dose around the PTV to spare the OAR. When the IMRT optimizations finished, the system shows four possible treatment plans: PTV only, where the optimization will only focus on the PTV coverage, and three OAR sparing optimization going from low OAR sparing until high, where the priority is given to the OAR. Depending on the results, one of this optimization was chosen. The objectives for target volumes and OARs were similar to the existing plans on helical Tomotherapy.

### RapidArc planning

RapidArc plans were also generated with the Trilogy (Varian, Palo Alto, CA) Linac equipped with the same high-resolution MLC model (Millennium 120 leaves MLC) as the one for the HybridArc plans. RapidArc plans consisted of two 358° arcs of 6 MV photon beam. To minimize the contribution of the tongue and groove effect during the arc rotation the collimator was rotated to ±20°. A RapidArc plan consists of optimizing a dose distribution from dose-volume objectives. Planning objectives were transferred into numerical dose-volume constraints used in the optimization phase and tailored to the specific patient characteristics. Priorities were adjusted during optimization to achieve the best results for each patient.

To achieve the required level of modulation, the optimiser is enabling to continuously vary the dose rate, MLC leaf positions and the gantry rotational speed. Plans were optimized selecting a maximum dose rate of 600 MU/min.

### Tomotherapy planning

Tomotherapy plans were generated using a fan beam thickness (FBT) of 2.5 cm. This choice was made because the larger available FBT of 5 cm resulted in a too-large penumbra in the cranio-caudal direction, resulting in more healthy tissue irradiated. Using the smaller available FBT of 1 cm resulted in an increase average overall treatment compared to the FBT of 2.5 cm. Pitch ranged from 0.287 to 0.31, depending on the level of difficulty to achieve the OAR constraints. Modulation factor varied from 2 to 3, depending on homogeneity and conformity index. Inverse planning consisting of numerical dose-volume constraints were used to optimize the plans to the specific patient characteristics. Priorities and dose penalties were adjusted during optimization to achieve the best results for each patient.

### Treatment planning evaluation

Several parameters of the dose volume histograms were computed to make the comparison between the different systems. For the PTV, the mean target dose and the volume receiving 95% of prescription dose were analyzed. Furthermore, the dose gradient towards the boost region (GI), defined as the ratio of the volume receiving more than 52.5Gy to the volume of the PTV55.2Gy, was analyzed. In concordance with the planning objectives on helical Tomotherapy, we tried to keep the GI in the HybridArc and RapidArc plans below 1.5. For the OAR, the mean dose and V15 for the small bowel and the mean dose for the bladder were analyzed. A comparison of delivery efficiency, such as beam-on time among the different treatment modalities was also conducted. As MUs cannot be compared in any meaningful way with helical Tomotherapy (fan-beam versus cone-beam), the MUs were only compared for the HybridArc and RapidArc plans. All the planning results were compared by a paired two-sided *t* test.

## Results

**Figure 2:**
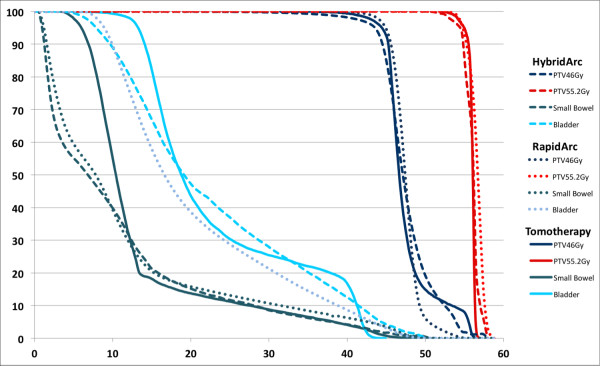
The average DVHs for the PTV46.0Gy, PTV55.2Gy, small bowel and bladder, for HybridArc (dashed-line), RapidArc (dotted-line) and Helical Tomotherapy (straight-line), respectively.

**Figure 3:**
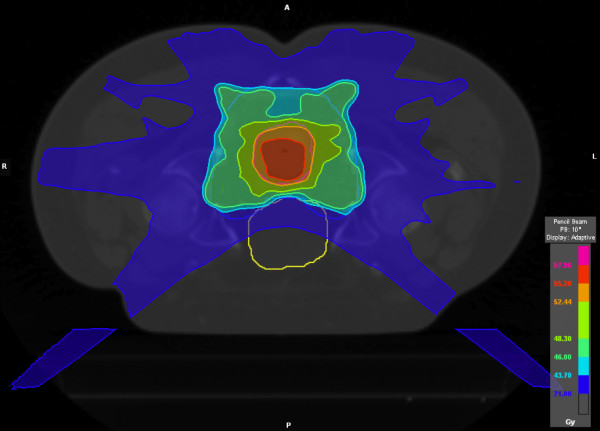
Axial view of typical dose distributions for HybridArc.

Figure 2 shows the average DVHs for the PTV46.0Gy, PTV55.2Gy, small bowel and bladder. The mean PTV volume was 1653.3cm^3^ (SD 225.0cm^3^) with a mean simultaneous-integrated-boost volume of 131.9cm^3^ (SD 95.8cm^3^). All treatment plans were able to achieve the constraints placed on PTV coverage. The mean doses to the PTV46.0Gy and PTV55.2Gy were 46.6Gy (SD 0.5Gy) and 56.2Gy (SD 0.4Gy) for HybridArc, 47.5Gy (SD 0.4Gy) and 56.5Gy (SD 0.3Gy) for RapidArc and 46.7Gy (SD 0.4Gy) and 56.1Gy (SD 0.4Gy) for helical Tomotherapy, respectively. The V43.7 (95% of 46.0Gy) and V52.4 (95% of 55.2Gy) were95.6% (SD 0.8%) and 99.1% (SD 0.5%) for HybridArc, 97.8% (SD 0.9%) and 99.3% (SD 0.5%) for RapidArc, and 96.5% (SD 1.7%) and 99.6% (SD 0.4%) for helical Tomotherapy, respectively. The dose gradients between the PTV46.0Gy and the PTV55.2Gy were steep for the different treatment modalities (p < 0.01) with a GI of 1.31 (SD 0.16), 1.28 (SD 0.10) and 1.33 (SD 0.16) for HybridArc, RapidArc and helical Tomotherapy, respectively. Typical dose distributions for HybridArc for one patient are shown in Figure 3 for an axial view.

Concerning the OARs, a significant advantage was found for helical Tomotherapy in sparing the small bowel (V15) compared to other solutions (HybridArc and RapidArc) (p < 0.01). The mean bladder dose was better spared RapidArc (p < 0.01). The mean small bowel dose was 11.5Gy (SD 3.9Gy), 11.3Gy (SD 3.8Gy)and 11.7Gy (SD 3.0Gy) for HybridArc, RapidArc and helical Tomotherapy, respectively. The V15 for the small bowel was 143.7cm^3^ (SD 74.4cm^3^), 133.4cm^3^ (SD 75.3cm^3^) and 112.7cm^3^ (SD 73.4cm^3^) for HybridArc, RapidArc and helical Tomotherapy, respectively. The wide SD for V15 of the small bowel is caused by the variability of the volume small bowel lying in the pelvis of the different patients. The mean bladder dose was 24.2Gy (SD 4.3Gy),20.6Gy (SD 2.2Gy) and 23.0Gy (SD 4.7Gy) for HybridArc, RapidArc and helical Tomotherapy, respectively.

For the treatment delivery efficiency, the mean beam-on time was significantly lower (p < 0.01) for HybridArc (2.7min SD 0.8) and RapidArc (2.5min SD 0.5) compared tohelical Tomotherapy (11.0min SD 0.7). The total amount of MUs was significantly (p < 0.01) higher for HybridArc (949SD 153) system compared to RapidArc (547 SD 44) system.

## Discussion

IMRT is mainly used to generate concave dose distributions, which are needed in the preoperative RT of rectal cancer to minimize the irradiated volume of small bowel and bladder. Especially, relative large PTVs enveloped by OAR such as those presented in rectal cancer have been proven to be challenging [15]. Techniques such as step and shoot IMRT, VMAT and helical Tomotherapy are able to create conformal isodoses with a reduction of the irradiated nearby healthy tissues [6,15]. Engels *et al.*[7] initially performed a dosimetric study comparing standard 3D-CRT with IMRT and showed a significant lower V15 of the small bowel and its NTCP for IMRT, while maintaining an adequate PTV coverage for the preoperative treatment of rectal cancer.

Moreover, IMRT allows dose escalation to the primary tumor without increased normal tissue toxicity with the simultaneous-integrated-boost strategy. Previous studies in our department explored this strategy in preoperative RT of rectal cancer by the use of helical Tomotherapy[6-8] displaying a favorable acute toxicity profile and no increased toxicity with the administration of a simultaneous-integrated-boost [6]. Differences in the characteristics of these IMRT techniques might lead to differences in target conformity, organ sparing (avoidance of toxicities to small bowel and bladder in rectal cancer patients treated with RT preoperatively), and treatment quality and efficiency.

Whenever a new treatment technique is introduced, it should be evaluated against well-established technologies. In the present planning study, we evaluated the feasibility of this novel treatment technique, HybridArc, for preoperative RT of rectal cancer and compared the planning results with respect to dosimetric parameters and the treatment efficiency to the helical Tomotherapy dedicated IMRT system, a technique in which we build up a wide expertise over the last years, and to RapidArc, a well-established VMAT solution used at the European Institute of Oncology (EIO) hospital in Milan. This planning study is to our knowledge the first evaluation of HybridArc, a novel automated direct-aperture optimized arc with equally distributed integrated IMRT-elements and demonstrated that HybridArc could achieve similar target coverage to well-established rotational IMRT solutions.

The GI showed a steep dose gradient between the simultaneous-integrated-boost and the PTV for the different planning systems. The OARs, more specifically for the V15 of the small bowel and the mean bladder dose, were less spared with HybridArc.

Several drawbacks of these techniques should be addressed. The complex process of treatment planning and treatment delivery requires extensive physics quality assurance [16]. Due to the completely dynamic implementation and new method of operating the Linac (dose rate and gantry speed changement during arc delivery), correct treatment delivery of VMAT must be extensively verified for both machine general specific performance and treatment plans [17]. The same has to be performed for helical Tomotherapy, due to its helical way of delivering dose and fan-beam irradiation. Therefore, for these treatment modalities, new Quality-Assurance (QA) schemes have to be elaborated. HybridArc starts from two well-known treatment modalities (DCA and Dynamic-MLC-IMRT (DMLC-IMRT)) and redesigned them at software base. The concept of decoupling the rotational delivery part (DCA) and IMRT-part place less burden on the mechanical constraints of the treatment machine. Because of this, there is no need to execute other specific performance on the LINAC and same dedicated QA schemes for DCA and DMLC-IMRT can be maintained to verify the treatment delivery. This study focused on comparing this new IMAT concept with a well known rotational IMRT concept (Tomotherapy) and a VMAT concept (RapidArc). Pre-treatment QA was beyond the scope of this study. Although, we can already anticipate as Petoukhova *et al.*[18] already evaluate the dosimetric accuracy for various treatment sites and found a good agreement for the HybridArc plans.

The prolonged beam delivery time of IMRT compared to 3D-CRT may worsen the accuracy of treatment due to increased intra-fractional patient motion [19]. Beam-on time of HybridArc is comparable to RapidArc, significantly lower as compared to helical Tomotherapy, which means faster treatment delivery without sacrifying quality of the plans. Moreover, as IGRT is used, treatments occurring in a shorter time after imaging may minimize the likelihood that treatment volumes stay beyond their respective treatment margins.

Another issue of concern is the increased number of MUs required in IMRT treatment, which leads to greater interleaf scatter dose hypothesizing an increasement in secondary malignancy induction [20]. Our data showed that HybridArc plans require 53% more MUs than RapidArc plans. The reason of this finding is that beside the single Aperture-optimized-Arcs, there are also 6 static IMRT elements, which require a large number of MUs.

Moreover, the risk of developing a second malignancy after RT is not only hypothesized on the scatter dose and MUs, but also on the volume of normal tissue receiving low-dose RT [20]. The improved OAR sparing and PTV coverage is achieved at the price of increased low dose exposure of the surrounding healthy tissue. helical Tomotherapy delivers dose circumferentially around the patient, potentially leading to an increase in the volume on normal tissues exposed to low doses, the effects of which are not well understood. Figure 2 showed that for HybridArc and RapidArc the low-dose wash to the OAR will be less compared to helical Tomotherapy.

## Conclusion

Although additional studies are needed to evaluate HybridArc for other anatomical sites, treatment plan QA and clinical outcome after HybridArc, this planning study demonstrated that HybridArc is a feasible technique for preoperative irradiation of rectal cancer patients. It was able to achieve a homogeneous PTV coverage in the simultaneous-integrated-boost scenario, while limiting the irradiated volume of OARs and keeping the beam-on time comparable to RapidArc and significantly lower than helical Tomotherapy. Compared to our standard in IMRT for rectal cancer, helical Tomotherapy, and a VMAT solution (RapidArc), HybridArc achieved similar PTV coverage, whereas it appeared to be less impressive in sparing the small bowel and bladder. The added value of HybridArc is that the treatment modality can be implemented on a LINAC that can neither change dose rate nor gantry speed during delivery of arcs. The only condition is that the LINAC is equipped with DCA and IMRT treatment techniques.By avoiding conflicting the mechanical constraints of the delivery system, the same LINAC performance and QA schemes for DCA and IMRT can be maintained.

## Competing interests

The authors are involved in an on-going collaboration with Brainlab AG.

## Authors’ contributions

Concept and design: TG, BE, CG, MDR. Acquisition of data: TG, CG, PDC, MD, TR, KT. Analysis and interpretation of data: TG, BE, CG, DV, MDR. Drafting of the manuscript: TG, BE, CG, MDR. Reading and approval of final manuscript: all authors.

## Author details

^1^Department of Radiotherapy, Universitair Ziekenhuis Brussel, Vrije Universiteit Brussel, Brussels, Belgium. ^2^Department of Medical Physics, European Institute of Oncology (EIO), Milan, Italy.
